# Development of Hybrid Piezoelectric-Fibre Optic Composite Patch Repair Solutions

**DOI:** 10.3390/s21155131

**Published:** 2021-07-29

**Authors:** Florian Lambinet, Zahra Sharif Khodaei

**Affiliations:** Department of Aeronautics, South Kensington Campus, Imperial College London, London SW7 2AZ, UK; florian.lambinet14@imperial.ac.uk

**Keywords:** composite patch repair, bondline integrity, PZT-FBG hybrid solution, guided waves

## Abstract

This paper proposes a hybrid structural health monitoring (SHM) solution for a smart composite patch repair for aircraft structures based on piezoelectric (PZT) and fibre optic (FO) sensors to monitor the integrity of a the bondline and detect any degradation. FO sensors are used to acquire guided waves excited by PZT transducers to allow the advantages of both sensor technologies to be utilised. One of the main challenges of guided wave based detection methodologies is to distinguish the effect of temperature on the propagating waves, from that of an existing damage. In this research, the application of the hybrid SHM system is tested on a composite step sanded repair coupon under operational condition (temperature variation) representative of an aircraft for the first time. The sensitivity of the embedded FO sensor in recording the strain waves is compared to the signals acquired by PZT sensors under varying temperature. A novel compensation algorithm is proposed to correct for the effect of the temperature on the embedded FO sensor spectrum in the hybrid set-up. The repaired specimen is then impacted with a drop mass to cause barely visible impact damage (BVID). The hybrid SHM system is then used to detect the damage, and its diagnosis results are compared to a PZT only based smart repair solution. The results show promising application of the hybrid solution for monitoring bondline integrity as well as highlighting challenges of the embedding of FO sensors for a reliable and repeatable diagnosis.

## 1. Introduction

Although composite structures are increasingly being used in aircraft structures, bonded composite repairs are still not certified on primary structures [1,2]. The main reason for this restriction is the lack of reliable techniques to verify the quality of a bondline after application, without destructive testing. In addition, it is challenging to achieve a consistent bondline quality because it is directly related to the skills of the technician. With the recent advances in manufacturing technologies, large scale composite parts are currently being manufactured in one shot for aircraft structures which means that when a primary part is damaged, if there is no possibility of repair, it has to be replaced which is not practical. The accepted practice for composite repair is bolted repair. Bolted repairs are heavy, introduce discontinuity in the fibre and generate stress concentration zone which can be the initiation of delamination. Therefore, many of the aircraft manufacturers and operators have directed their research to finding a solution for composite bonded repair and addressing challenges related to the bondline integrity.

Structural Health Monitoring (SHM) techniques offer a potential solution for bondline integrity monitoring. SHM techniques have been extensively developed and tested for detecting external impact events through passive sensing methodologies [3,4,5,6,7,8] and consequently barely visible impact damage (BVID) through active sensing techniques [9,10,11,12,13,14]. Two of the main sensor technologies that have received attention are Piezoelectric (PZT) transducers and Fibre optic (FO) sensors due to their many advantages, either used individually [15,16] or in combination [17,18,19,20,21,22]. There are other sensor technologies for bondline monitoring which have been recently developed [23,24,25] and hence have lower maturity than PZT and FO sensors, in particular, in harsh operational environments such as aircraft structures. In a hybrid system the advantages of utilising PZTs as actuators is combined together with the advantages of the FO sensors which have high sensitivity to strain, low weight, ability to compensate temperature, possibility of multiplexing and immunity from electromagnetic interference [20,26].

In the particular case of a composite patch repair, fibre optics are easier to embed in composites than PZT transducers [27] and have been proven to be successful in detecting damage [28,29]. The disbond detection of adhesive layers has been previously reported with Fibre Bragg Grating (FBG) sensors for a honeycomb core and facesheet [30] and single lap joint [31]. In other work, the evolution of disbond of the adhesive layer in composite repair patches has been successfully monitored [32]. However, the FBG sensors were used solely as strain sensors, leading to good damage detection only if the damage is close to the sensors and the structure is not under a variable load. Very little research can be found on monitoring of a step-sanded patch repair which poses a much more challenging structure than a lap joint. In the authors previous work [33], a network of PZT sensors was used for monitoring of a step sanded patch repair. However, it was noted that the transducers could only be surface mounted, which resulted in lower sensitivity to detecting changes in the bondline. Moreover, surface mounted transducers have the challenge of having wires and connectors on the surface or surrounding the patch, which can interfere with the functionality of the structure.

In this paper, a hybrid PZT-FBG monitoring system is developed and tested for the first time, for a bonded composite step-sanded repair for an aircraft structure with embedded FBG sensors in the bondline. The proposed smart repair solutions address the challenges related to the quality assurance of bonded repairs on primary structure. In a previous work, the feasibility and the challenges of guided wave based SHM with PZT transducers on composite patch repair has been reported and demonstrated [33]. This work extends the developments to fibre optic (FO) based techniques (FO as passive sensor and in combination with guided waves to form a hybrid system). Section 2 first introduces the composite patch repair strategy to highlight the challenges related to its application to primary structures. This section also introduces the state of the art and the potential application of SHM to composite bonded repair, before the details of the hybrid PZT-FO system are outlined in Section 3. Since there is no commercial off-the-shelf hardware/software set-up available for hybrid PZT-FBG application, the development of the system is detailed. The feasibility of a hybrid system capable of monitoring complex structure under aircraft operational environment is demonstrated with an experiment outlined in Section 4. The test structure is a step-sanded composite patch repair where FBG sensors are embedded in the adhesive layer of the patch. Environmental variations representing in-service conditions of aircraft, especially temperature, are applied to the SHM system in order to evaluate their applicability in service. The results of FBG sensors alone and in combination with PZT transducers are discussed in detail in Section 5, followed by conclusion in Section 6 where the potential and challenges of such smart repair solution that rises by utilising FBG sensors for recording ultrasonic guided wave are summarised.

## 2. SHM for Composite Patch Repair

There are three typical bonded composite repair types: patch repair, scarf (or tapered) repair and step-sanded repair. The tapered (scarf) repair and the step-sanded repair are the most difficult ones to achieve and require specialised equipment making them hard to manufacture on field. On the other hand, they achieve the best properties in strength and fatigue resistance; hence, they are mostly used for structural repairs while patch repairs are preferred for cosmetic/temporary repairs. The tapered/step-sanded repairs are flush, leading to better aerodynamic performance for external repairs and also the over-peel stresses induced by eccentricity are low. In order to increase the level of certainty on the bondline of bonded repairs, OEMs and MROs have found solutions where the human factor is as small as possible using automation in order to achieve better repeatability and overall quality of the repairs e.g., a portable waterjet cutter called the Repair Jet developed by Airbus. One solution to certify the quality of the bondline is using Bonded Repair Coupons (BRCs) [34]. The BRCs are bonded simultaneously with the repair patch; hence, their cure conditions are equivalent. Subsequently, a simple and practical torque test on the BRCs can then be performed validating the quality of the repair bond. However, they still face disadvantages such as contamination of the joints and aero-dynamical disturbance to the main structure. SHM has the potential to monitor the integrity of the structure remotely and increase the reliability of diagnosis by providing continuous monitoring during its service life, [9,10,11,12,13]. SHM’s application to bonded composite parts can address the challenges related to the strict quality assurance of the bonding. The bond quality cannot be guaranteed with conventional NDT techniques, and the presence of dry contact kissing bonds is difficult to detect with traditional ultrasonic techniques [35]. The concept of ’smart patch’, a composite patch repair with in situ sensors that can assess its own structural health, was proposed at the end of the 90’s for military aircraft. [36]. Two autonomous solutions were developed [37], and the disbonds were monitored using strain measurements utilising strain gauges and PVDF sensors. Magnetostrictive sensors can also be used to monitor repair integrity when embedded in the adhesive line of the repair [38]. There are early examples of Fibre Bragg Gratings (FBGs) sensors applied to successfully monitor the health of the bonded composite patch repairs [39,40,41] if placed near the bondline or embedded. Piezoelectric transducers have been used as well, first through their impedance response to determine the patch integrity [42,43], followed by transmitting and receiving ultrasonic Lamb waves [44,45]. These earlier examples were concerning metallic structures repaired by bonded composite patch. With the arrival of composite structures, acousto-ultrasonic SHM techniques, while being an alternative [46], became more popular [47]. An example of Lamb waves based SHM can be reported in monitoring the debonding of composite patch repair of a vertical helicopter stabiliser with a pre-introduced crack under fatigue [48]. The health monitoring of composite repair has been also achieved with sensors placed in ’hotspots’ where the likelihood of disbond is high [23,49]. However, the current SHM solutions, lack reliability, repeatability and are sensitive to environmental effects. There is also little research reported on their application in monitoring of complex structural repair such as a step-sanded repair where the ply drop-off can be a hotspot for initiation of delamination/debond.

This paper investigates the applicability of a hybrid SHM technique to a step-sanded composite bonded repair under operational conditions, such as varying temperature, for the first time. The proposed smart repair solution based on hybrid FO-PZT system is developed with embedded FO sensors in a circular pattern to follow the geometry of the bondline, tested and verified experimentally in this work. The challenges that are addressed in this work which represent progress beyond the state of the art are the embedding of FO sensors in the bondline of a step-sanded repair, the development of reliable software/hardware for acquiring guided waves with multiplexed FBG sensors under operational conditions (varying load and temperature), and finally, development of a reliable calibration algorithm to compensate for the effect of varying temperature and load.

## 3. Hybrid PZT-FO Data Acquisition System

**Figure 1:**
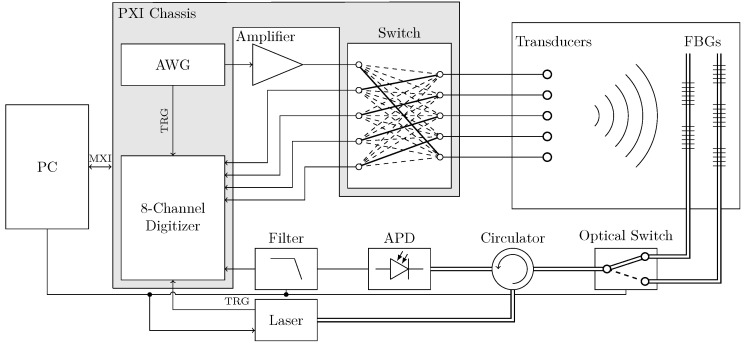
Hybrid acquisition diagram.

Multiple Fibre Bragg Grating (FBG) sensors can be engineered on a single fibre each with different wavelength. FBG sensors are very attractive strain sensors due to their sensitivity με, having high signal-to-noise ratio (SNR) and being an attractive choice for detecting small changes in the propagation of guided wave signals which can be used for damage detection. However, there are several parameters in the acquisition procedure that are crucial in developing a reliable system. The data acquisition needs to be completely automatic since the proposed SHM system is a remote maintenance solution which does not require access to the part. This section covers the hybrid data acquisition, different instruments, their interactions and their respective specifications as summarised in Figure 1 and Table 1.

### 3.1. Optimum Focus Wavelength

**Figure 2:**
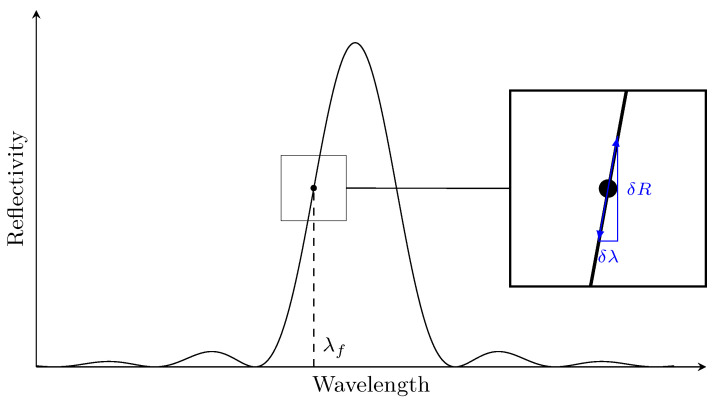
FBG sensor conversion.

In theory, strain and temperature induce a proportional shift in the Bragg wavelength of the FBG sensors. This hybrid acquisition system can only focus on one wavelength in order to measure the reflectivity amplitude change. The window where the focus wavelength is effective is very narrow (around 0.2 nm). In order to prevent the sensing wavelength missing this window because of temperature variation (10 ${}^{{}^{\circ}}C$ temperature change leads to approximately 0.1 nm wavelength shift), an optimal focus wavelength or ‘focus point’ selection algorithm was developed to determine the optimal focus point $\lambda_{f}$, as shown in Figure 2, chosen to be the point with the highest reflectivity derivative between 25% and 80% of the peak amplitude. The limits have been set to avoid any shape change at the base and top of the peak. The highest reflectivity derivative is used to maximize the reflectivity amplitude change, thus increasing signal-to-noise ratio.

When the focus points are determined for each FBG sensor, the Lamb wave acquisition can start. The tunable laser will be set to a constant focus wavelength of the FBG sensor, and the digitiser records the filtered reflectivity change at that focus point. The Arbitrary Waveform Generator (AWG) triggers the digitiser in order to synchronise the two instruments. Different actuation signals have been used in order to study the effect of frequency on damage detection: 5 cycle Hanning window actuation signal with frequencies from 50 to 300 kHz at 25 kHz interval, 60 V amplitude.

### 3.2. Calibration Method

Although the measurement of Lamb waves by FBG sensor has a few advantages such as higher strain sensitivity and electromagnetic interference immunity, the effect of environmental factors such as temperature has to be taken into account for a rosbust diagnosis. Although there have been some developments in baseline free damage detection techniques based on Guided waves [58,59,60,61], the mature methodologies rely on comparing the current state of the structure with a pristine baseline. Therefore it is crucial that both states (pristine and current) do not have any variations in the recorded signal that are due to changes in the environmental and operational conditions of the structure, which subsequently can be mistaken for damage. Temperature variation not only affects the piezoelectric properties of the transducers and the Lamb wave propagation but also the reflectivity behaviour of the FBG sensors by shifting their Bragg peak and changing its shape which influences the FBG sensor strain sensitivity (conversion between reflectivity change to strain change). This scaling issue can be addressed by normalising the signals, which results in losing the strain amplitude information, which is essential in damage detection. This section describes a novel post-processing calibration method that converts the hybrid Lamb wave reflectivity signals received by the acquisition system into strain readings, thus removing the temperature bias of the FBG sensors but also compensating the effect of temperature on Lamb wave propagation. This calibration procedure increases the robustness of the hybrid SHM systems in detecting damage under changing environmental conditions.

#### 3.2.1. Strain Conversion

**Figure 3:**
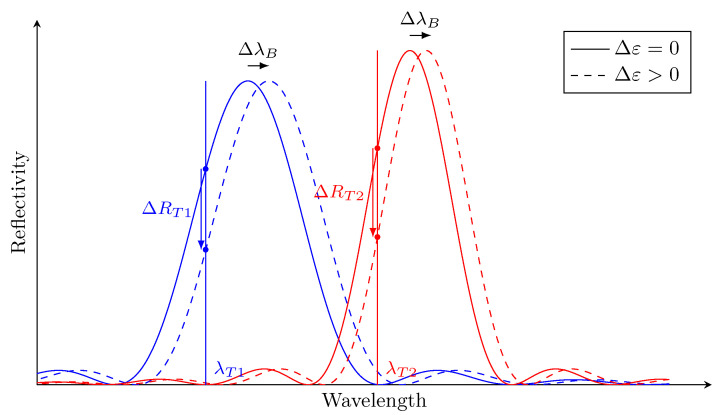
Reflectivity changes due to temperature for the strain change Δε at T1 (blue) and T2 (red).

Even if the sensing wavelength $\lambda_{f}$ is determined dynamically for every temperature, the strain change is measured by a variation of the reflected light power. However, the reflected light power change $\Delta R_{T1}$ at the temperature T1 induced by a Bragg peak shift (due to strain Δε) $\Delta \lambda_{B}$ is not equal to the reflected light power change $\Delta R_{T2}$ at a different temperature T2 induced by the same Bragg shift (see Figure 3). This difference is due to the fact that temperature changes the reflectivity of the peak due to non-uniform strain fields at the FBG location. The overall effect is an amplitude mismatch between two Lamb wave signals measured at different temperatures, which can be mistaken for damage. In order to completely remove this effect, every hybrid Lamb wave signal is converted to its equivalent strain recording instead of being used as a change of reflectivity as is the current practice. Although the wavelength peak shift δλ is proportional to the strain change δε (the linear coefficient is often called sensor gauge κ), the variation of reflected light power δR depends on the slope of the Bragg peak (as shown in Figure 2). This slope is not constant and differs for each FBG sensor and if not treated carefully, it leads to scaling problems: reflectivity signals from different operational conditions (load, temperature) or FBG sensors are scaled differently because of the slope at $\lambda_{f}$. In order to ensure that the reflectivity of the hybrid signals are comparable to each other even from different FBGs, they have to be converted to relative displacement or strain.

Because the Lamb wave induced wavelength shift is relatively small (pm), the derivative of the reflectivity spectrum is considered constant at the point of focus $\lambda_{f}$, leading to

(1) $$R^{\prime }\left(\lambda_{f}\right)\approx \frac{\delta R}{\delta \lambda }$$

by substituting the linear wavelength shift δλ due to strain (κ=1.2 pm/µϵ)

(2) δλ=κ·δε

into Equation (1) the strain change can be expressed by

(3) $$\delta \varepsilon ={\left(R^{\prime }\left(\lambda_{f}\right)\;\cdot \;\kappa \right)}^{-1}\delta R$$

leading to the conversion factor $R_{conv}$

(4) $$R_{conv}={\left(R^{\prime }\left(\lambda_{f}\right)\;\cdot \;\kappa \right)}^{-1}$$

At a given temperature, each FBG sensor *i* has a different $R_{conv}^{i}$ that needs to be calculated from their reflectivity spectrum. Following this, the hybrid signals recorded from the reflectivity light power (in V) can be converted to comparable dynamic strain measurements (in µϵ).

#### 3.2.2. Temperature Compensation

While the conversion step ensures hybrid paths are comparable to each other, it does not remove the effect of temperature on the Lamb wave propagation. As it has been demonstrated [62], the effect of temperature variation on the propagated wave is change in amplitude and phase, depending on the material properties of the host and the frequency of the travelling wave. In order to correct of the amplitude, the damage signals are scaled to their reference or pristine counterparts. This step removes the amplitude shift due to temperature and also greatly reduces the mismatch error on the conversion factor $R_{conv}$. In practice, only the reference recordings are converted while the damage signals are scaled. Due to the multi-modal nature of Lamb waves in composite structures and especially composite patch repairs, the phase of the damage signals $S_{T_{Ref}}^{Damage}$ is also aligned to the reference $S_{T_{Ref}}^{Ref}$ with Instantaneous Phase Alignment (IPA):

(5) $$S_{T_{Ref}}^{Damage}=ℜ\left(S_{T}^{Damage}e^{i(\phi_{Ref}-\phi_{Damage})}\right)$$

where

(6) $$\phi_{Damage}=\arg\left(S_{T}^{Damage}+iH\left(S_{T}^{Damage}\right)\right)\phi_{Ref}=\arg\left(S_{T_{Ref}}^{Ref}+iH\left(S_{T_{Ref}}^{Ref}\right)\right)$$

**Figure 4:**
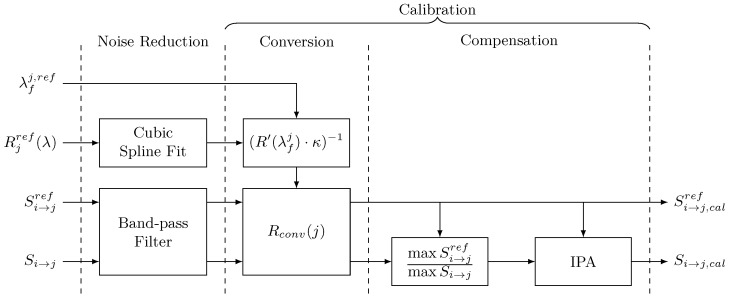
Compensation block diagram.

The scaling and IPA procedures should reduce the effect of temperature while not compensating the effect of damage between the pristine and damage signals. The overall calibration process used in this study is shown in Figure 4.

After a de-noising procedure for both the hybrid signals and the reflectivity spectrum, the conversion is applied to both pristine and damage signals. The conversion factor $R_{conv}$ is obtained from the pristine sensing wavelength and FBG reflectivity spectrum. The different paths are converted with their respective FBG sensor conversion factor. Once the hybrid signals have been converted to relative displacement, the damage signals receive additional processing and are compensated for temperature effects.

## 4. Experimental Set-Up

In order to test the hybrid smart repair solution, one PZT/FBG configuration was chosen with a similar number of transducers and FBG sensors: a composite step-sanded repair was equipped with five surface-mounted piezoceramic patch transducers and five FBG sensors embedded in the bondline of the last repair ply where disbonds are most likely to occur. In order to study the effect of environmental conditions on the sensors and later on damage detection, the pristine and damaged state measurements were recorded over a range of temperatures. The damaged state has been created by impacting the repair and successfully introducing a BVID.

### 4.1. Repair Manufacturing and Sensorising

**Figure 5:**
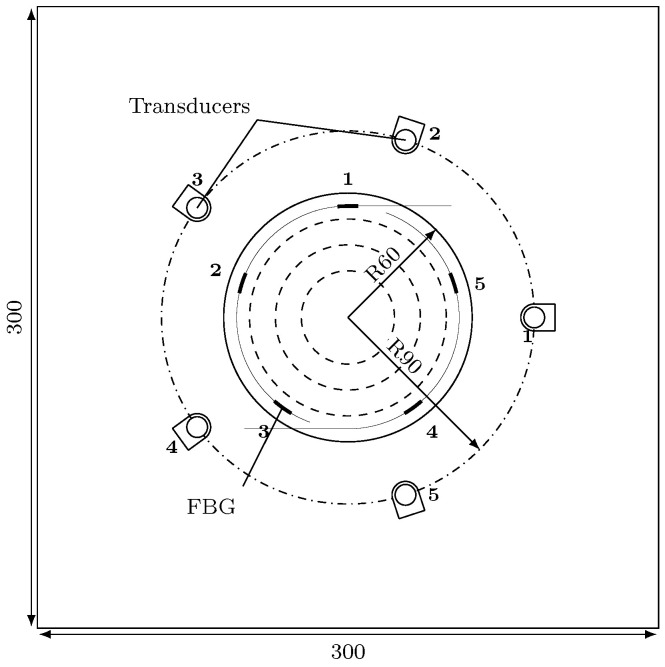
Specimen schematic.

**Figure 6:**
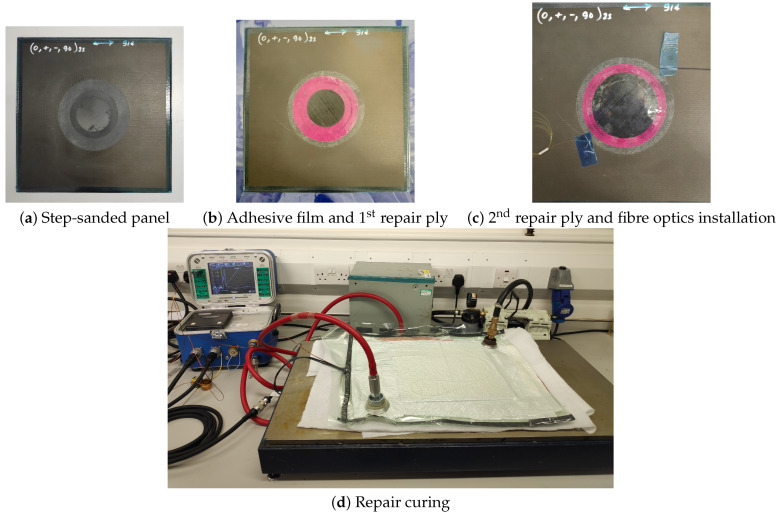

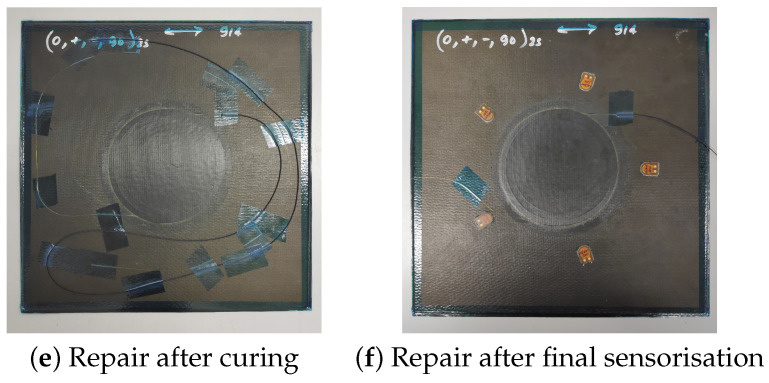
Specimen preparation, repair and sensorisation steps.

The 300 mm × 300 mm × 2 mm panel is made of sixteen T300/914 prepreg plies with a [0/+45/−45/90]${}_{2s}$ quasi-isotropic lay-up. The ϕ120 mm step-sanded repair is made of three T300/914 plies with an overlap length of 12.5 mm and a Cytec FM300 adhesive film (0.25 mm). Two fibre optics with three and two FBGs respectively have been inserted in the adhesive film as shown in Figure 5, evenly distributed on a circle of radius 60 mm. The five FBG sensors are manufactured by Micron Optics (os1200). They have been cut to dimensions and spliced together. Their direction of entry in the repair was chosen to be aligned with the 0${}^{{}^{\circ}}$ fibre direction of the last layer to minimise the ingress and egress stresses on the fibre which could cause the fibre to break. The repair has been cured in an autoclave. The repaired panel was then equipped with five PZT transducers (DuraAct manufactured by PI ceramics) surface mounted with thermoplastic film. The transducers were positioned around the repair evenly distributed on a circle of radius 90 mm. Figure 6 shows different stages of the repair preparation and sensorization.

### 4.2. Temperature Variations

The repair remains on the structure which undergoes operational and environmental variations during flight and also on the ground. The robustness of the hybrid damage detection under environmental effects needs to be investigated as the Lamb waves and FBG readings are to be recorded on the field. Each state of the structure is recorded at different environmental conditions after each flight. In the case of temperature variations, the aircraft is considered to operate in the European region. According to MIL-STD-810G [63], most of Europe has a ’Basic’ climatic design type (Basic Cold C1, Basic Hot A2), and the ambient air minimal temperature should be above −31.7 ${}^{{}^{\circ}}$C (−25 F), and the maximum temperature should be below 40.6 ${}^{{}^{\circ}}$C (105 F). The temperature chosen for this study is (−20 ${}^{{}^{\circ}}$C, 40 ${}^{{}^{\circ}}$C). The pristine and damage(impacted) states were recorded inside the J2235 Thermal Vacuum Chamber supplied by Temperature Applied Sciences LTD. The temperature of the chamber was varied between −20 ${}^{{}^{\circ}}$C and 40 ${}^{{}^{\circ}}$C in steps of 5 ${}^{{}^{\circ}}$C.

### 4.3. Impact Test

**Figure 7:**
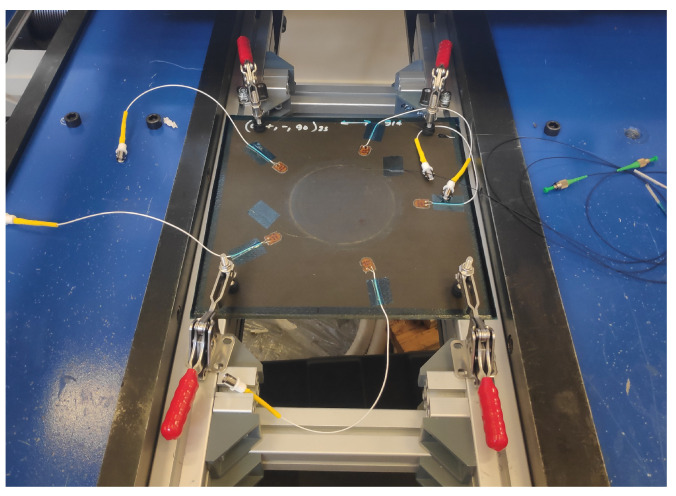
Impact test, clamped specimen.

The BVID was introduced on the composite patch repair with a low-velocity impact. An Instron CEAST 9350 drop tower was used to impact the top of the panel with a 10 mm-radius hemispherical impactor. The impact energy was set to 14 J, and the specimen was clamped as shown in Figure 7. C-scans from a portable DolphiCam CF16 Ultrasound Camera before and after the impact confirmed that the impact introduced a BVID.

**Figure 8:**
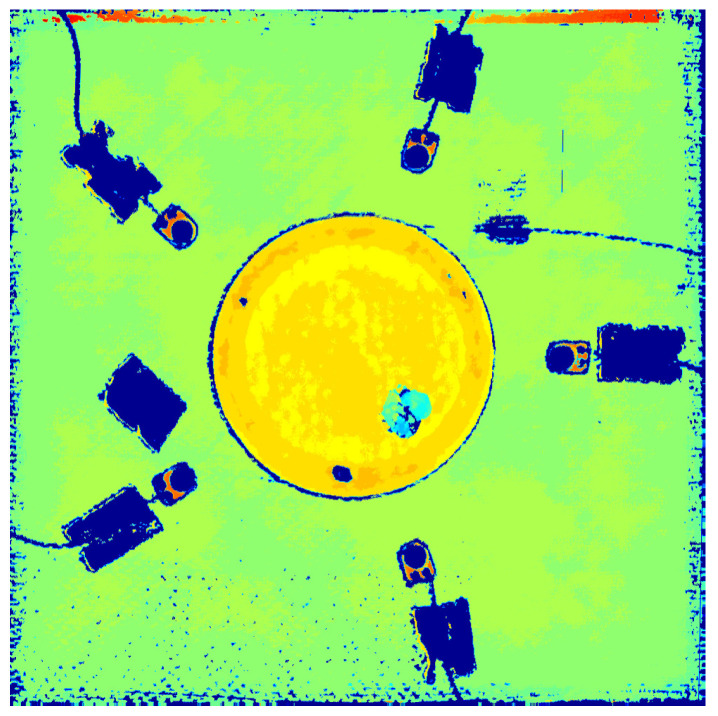
C-scan, after impact.

The specimens were fully scanned afterwards to verify the presence of the damage and inspect the overall health of the repair with embedded FBGs (Figure 8).

## 5. Results and Discussion

Throughout the experiment, two different states of the hybrid repair were recorded: before and after impact. For each state, the hybrid system was interrogated for thirteen different temperatures ranging between −20 and 40 ${}^{{}^{\circ}}C$. The hybrid system has the capability to use two different measurements: reflectivity spectrum (FBG) and hybrid signals (PZT→FBG). The results are therefore divided into two main categories. Firstly, the effects of temperature on the FBG sensors are presented, and the impact detection using only FBG sensors is investigated. Secondly, the study focuses on the robustness of the proposed calibration method to reduce the effect of temperature while maintaining the sensitivity of the detection algorithm to impact damage. Finally, the damage detection capability of the calibrated hybrid system is assessed.

### 5.1. FBG Sensors Only

#### Reflectivity Spectrum under Temperature Change

**Figure 9:**
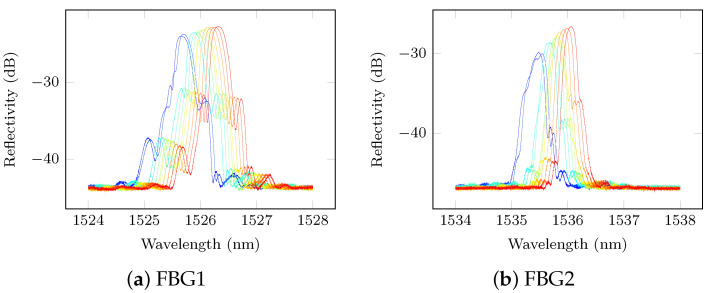

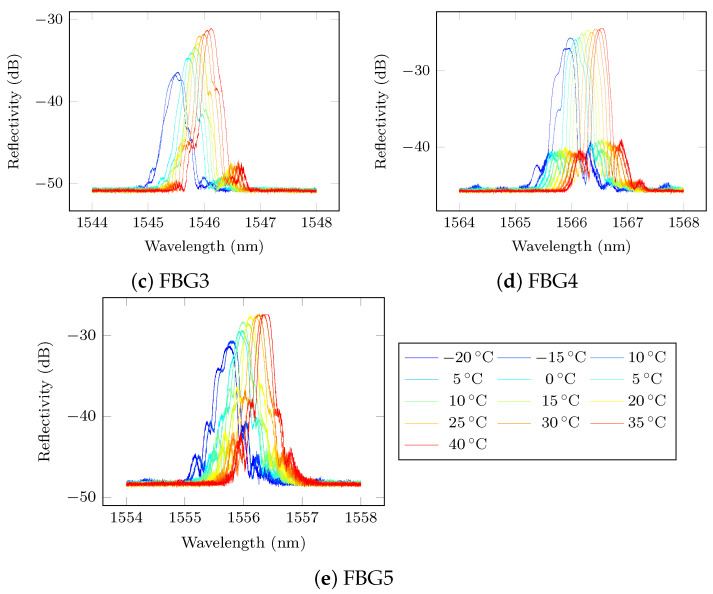
Reflectivity Spectrum under temperature changes, pristine state.

Since the five FBG sensors are distributed amongst two fibre optics following a circular pattern, spliced and embedded inside the bondline of the repair, their reflectivity is different and difficult to predict under temperature variations. Figure 9 shows the reflectivity spectrum (in dB) for each FBG from −20 to 40 ${}^{{}^{\circ}}C$. It can be seen that their reflectivity changes with temperature differently since each sensor has different orientation and different losses due to splicing.

**Figure 10:**
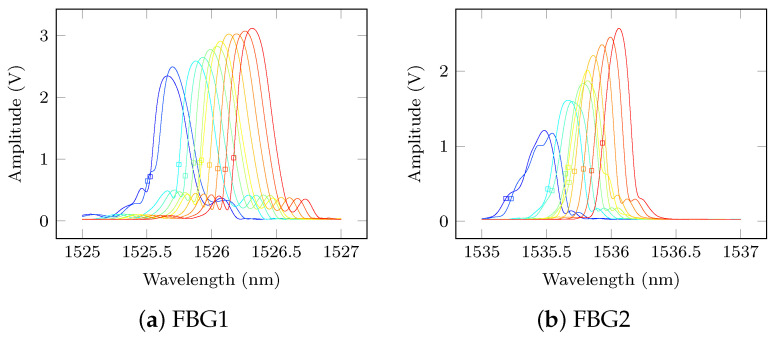

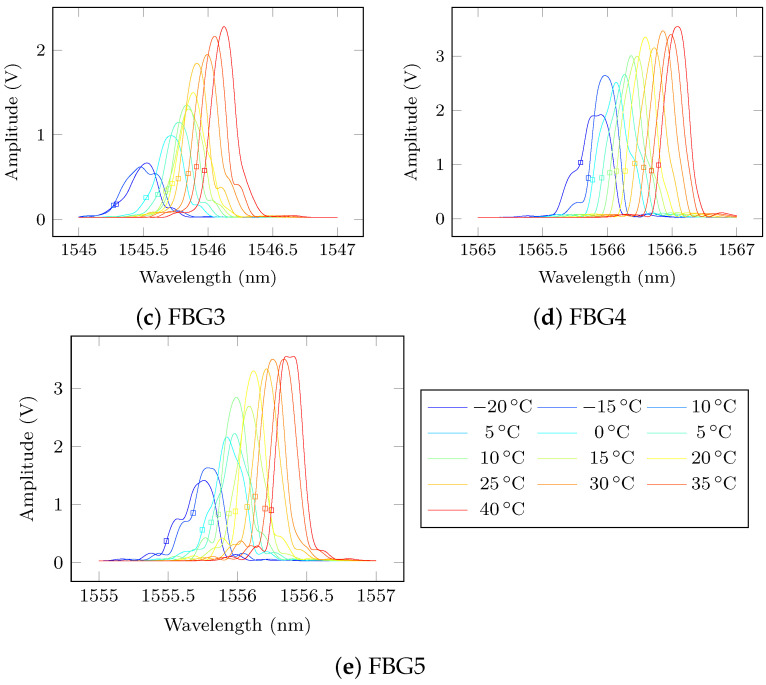
Sensing wavelengths used during the data acquisition procedure, pristine state.

However for all FBGs, the shift of the Bragg peak due to temperature is observed. Another interesting observation in every FBG reflectivity spectrum is that their maximum amplitude increases with temperature. In particular, FBG 2-5 exhibit a change in shape that could be explained by the non-uniform temperature distortion of the FBG arc (most of FBG sensors are used along their length in a straight line). These changes to the Bragg peak make the focus point selection challenging if no calibration were applied. Fortunately, the proposed calibration method has successfully corrected for the sensing wavelength optimal selection as shown in Figure 10 where the reflected Bragg spectrum is displayed in amplitude (V) and has been de-noised with a cubic spline.

**Figure A1:**
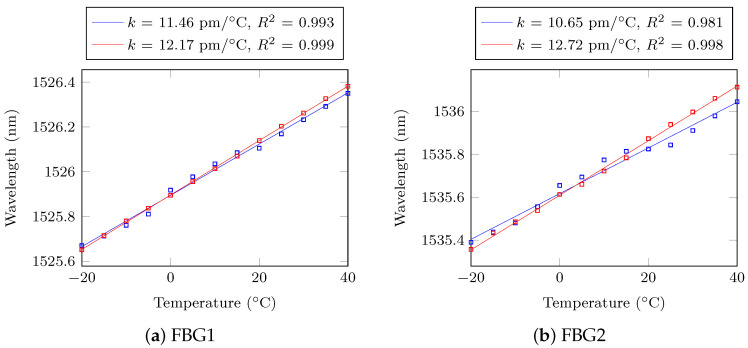

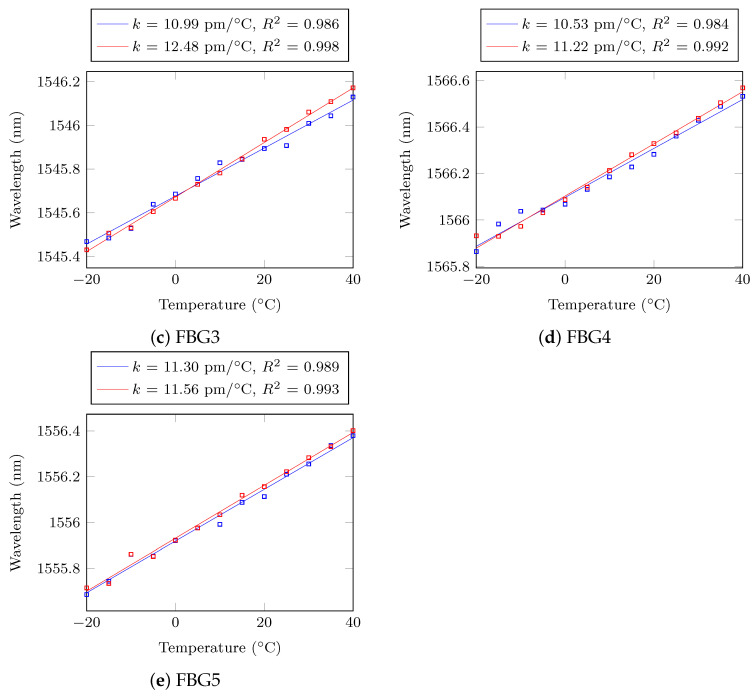
Bragg Wavelength peak under temperature change, pristine (blue) and impacted (red) states.

In order to verify that the Bragg peak shift due to temperature is still linear, the centre of the Full Width Half Maximum (FWHM) bandwidth was used to calculate the Bragg wavelength (X-dB Bandwidth technique, 3 dB [64]). This technique has a better accuracy than usual peak detection because of the changes in shape previously described leading to inaccuracies. The Bragg peaks calculated at each temperature before and after result in a linear thermal response coefficient *k* (pm/${}^{{}^{\circ}}C$), for every FBG was calculated using linear regression (it corresponds to the slope). Results are displayed in Figure A1 in Appendix A.

### 5.2. Hybrid Lamb Waves

The effects of damage on propagating Lamb waves recorded by FBGs are expected to be similar to the signals recorded by PZT only system [33]. However, the FBG sensors have completely different strain sensitivities which needs to be considered. While the circular piezo-ceramic transducers are sensitive to in plane strain from all directions, the FBG sensors are only sensitive to the strain along the fibre axis. Furthermore, the transducers are surface-mounted and the gratings are embedded under the top ply; hence, the acquired Lamb wave signals are expected to be significantly different.

**Figure 11:**
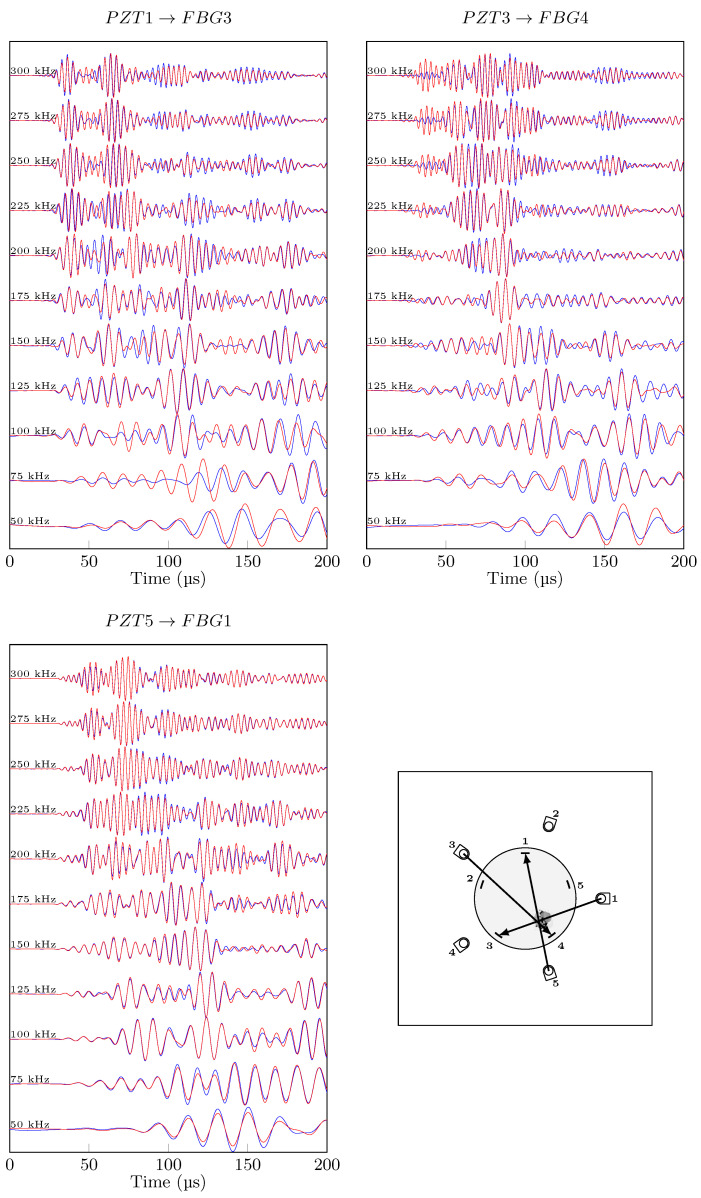
Different direct hybrid paths, pristine (blue) and impacted (red) states at 20 ${}^{{}^{\circ}}C$.

**Figure 12:**
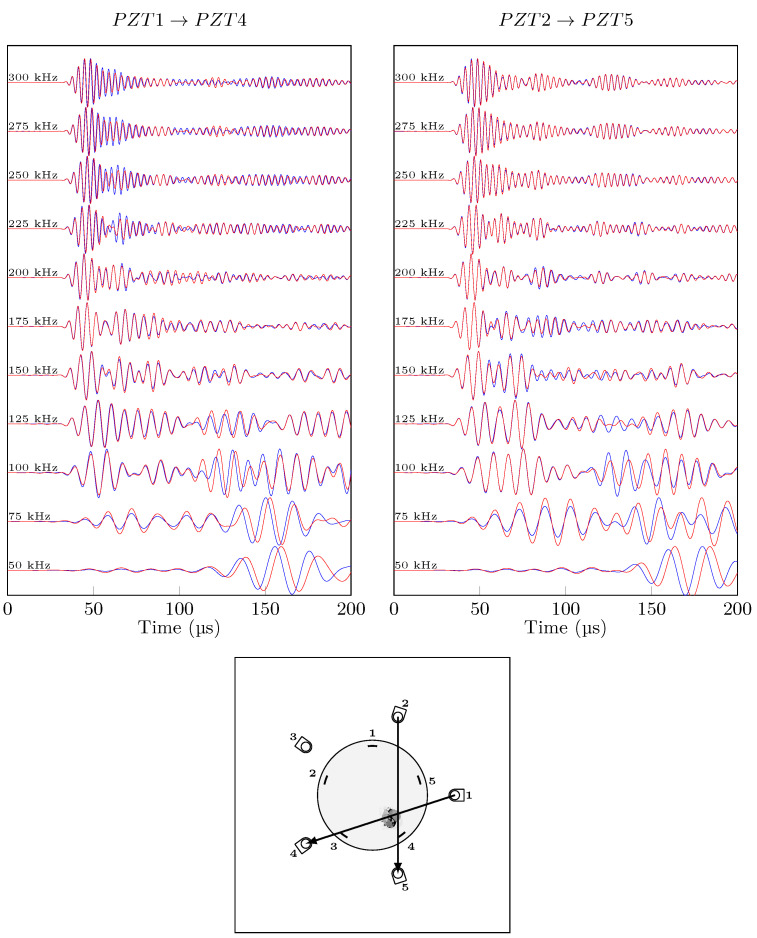
Different direct conventional paths, pristine (blue) and impacted (red) states at 20 ${}^{{}^{\circ}}C$.

In order to qualitatively compare the effect of damage on Lamb wave signals for conventional and hybrid paths, the same actuation signal was excited, and the different frequency responses are plotted in a waterfall plot in (Figure 11 for the hybrid set up and Figure 12 for PZT only acquisition i.e., conventional), for pristine vs. impacted signals with no temperature variation. The differences between conventional and hybrid signals are significant: while the conventional signals for two visualised paths can be considered similar in mode shape and time or arrival, the hybrid paths are different. At low frequencies (50 kHz), A_0_ mode in conventional paths is very sensitive to damage with a notable phase shift on both direct paths through damage (PZT3→FBG4 and PZT2→FBG5) while at high frequencies, the effect of damage is less pronounced on the anti-symmetric mode. On the other hand, the hybrid signals are different from each other, which is due to different fibre orientation, different central wavelength and different reflectivity properties. The path PZT3→FBG4 shows noticeable changes due to damage for the first wave-packet (S_0_) for the whole frequency range. Path PZT5→FBG1, on the other hand, has a slight increase in amplitude for A_0_ mode due to damage, at high frequencies only. Finally, Path PZT1→FBG3 displays good sensitivity to damage with both S_0_ and A_0_ modes. A_0_ seems to be prone to phase shift with the presence of damage, while amplitude change mostly appears for S_0_. Conventional and hybrid guided waves techniques have both different advantages and drawbacks for detecting damage in composite patch repair:

- PZT→PZT guided wave recordings sensitivity to damage is great at low frequencies, and it does not depend on the direction of propagation, but it is lower than hybrid.
- PZT→FBG guided wave recordings, depending on the propagation angle, shows greater sensitivity than its counterpart for S_0_ mode at any frequencies. Angle dependency is problematic for damage localisation purposes.

However, the results discussed so far have been in a controlled environment with no temperature variation. Next, the capabilities of the proposed calibration method under varying temperature are investigated.

### 5.3. Calibration Performance

First, the effect of the strain conversion on the hybrid measurements is demonstrated. Then, the robustness of the calibration procedure to temperature variations and its effect on damage detection is evaluated. It is important to demonstrate that the calibration procedure is capable of removing the effect of temperature from the recorded signal, while it does not correct for the effect of damage. Therefore, first the strain conversion is demonstrated on pristine signals, followed by its capability in damage detection under various temperature.

#### 5.3.1. Strain Conversion

**Figure 13:**
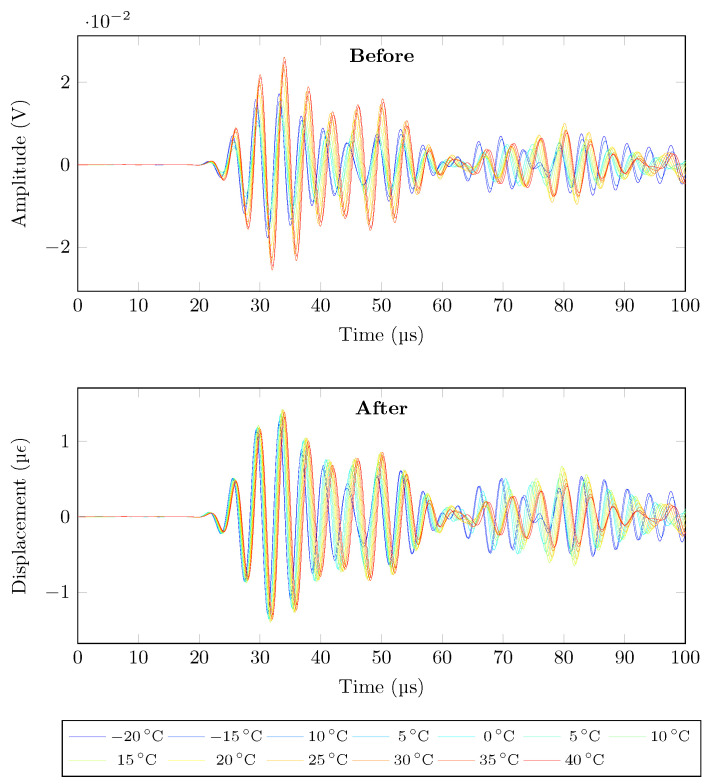
Strain conversion effect, PZT1→FBG1, 60 V 250 kHz, pristine state.

The proposed strain conversion eliminates the effect of the difference in reflectivity measurement. This is fundamental for any baseline comparison damage detection since it will result in difference in amplitudes for each acquisition. As shown in Figure 13, for signals of the path PZT1→FBG1, there is a significant amplitude change due to temperature variation resulting from different strain conversion factor. If not corrected, it will result in false alarm. After the strain conversion under temperature change, it is evident that only the effect of temperature on the propagating wave has been recorded.

#### 5.3.2. Temperature Change

**Figure 14:**
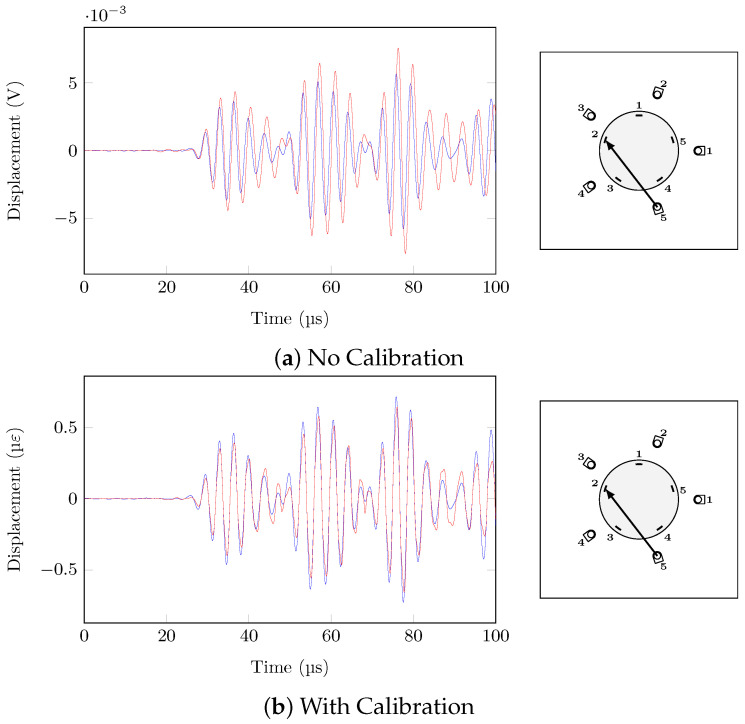
PZT5→FBG2, 60 V 300 kHz, pristine states, T = 20 ${}^{{}^{\circ}}C$ (blue) and T = 35 ${}^{{}^{\circ}}C$ (red), ΔT = 15 ${}^{{}^{\circ}}C$.

Path (PZT5→FBG2) has been selected in order to showcase the effect of the proposed calibration methodology (strain conversion and temperature compensation) on the hybrid signals under temperature variations with and without presence of damage, which will be discussed next. In the case of temperature change and no damage, the two pristine states at different temperatures (20 ${}^{{}^{\circ}}C$/35 ${}^{{}^{\circ}}C$) should be similar after the calibration procedure in order to avoid false alarms due to temperature variations. Figure 14 shows the effect of the calibration on the two signals at different temperatures, with the signal at 20 ${}^{{}^{\circ}}C$ being considered as the reference. While the signals are not completely superimposed, the calibration procedure completely removed the phase change and reduced drastically the amplitude change due to temperature variations.

#### 5.3.3. Damage and Temperature Change

**Figure 15:**
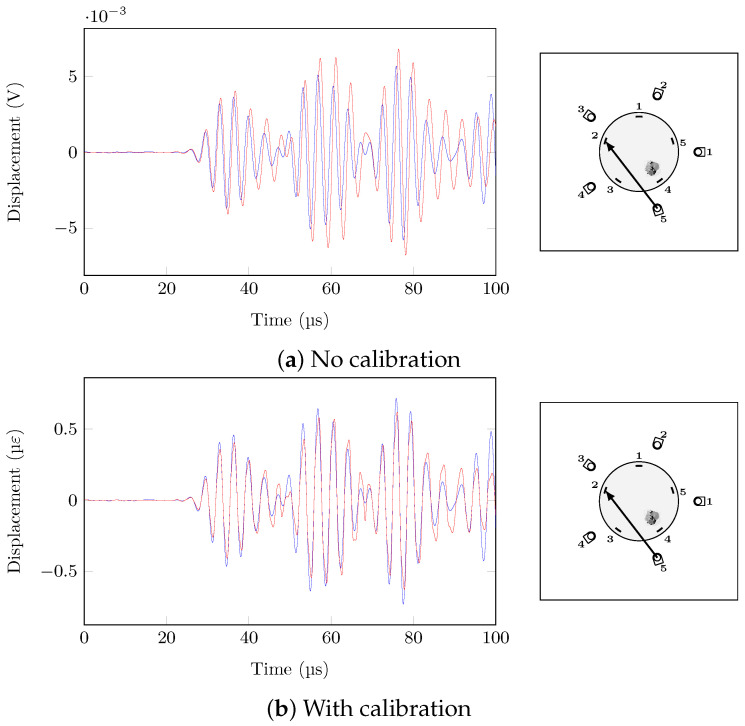
PZT5→FBG2, 60 V 300 kHz, pristine (T = 20 ${}^{{}^{\circ}}C$, blue) and damage (T = 35 ${}^{{}^{\circ}}C$, red), ΔT = 15 ${}^{{}^{\circ}}C$.

The real challenge is to reliably detect damage under varying temperature, i.e., correct the effect of temperature change but not the damage effect. For a ΔT of 15 ${}^{{}^{\circ}}C$, the effect of damage is really difficult to identify without calibration because its intensity is negligible in comparison to the effect of temperature, as shown in Figure 15. On the other hand, with calibration, the temperature change is greatly attenuated, but the damage effect is partially compensated.

The beneficial effects of the calibration procedure cannot be measured by only focusing on one path as damage detection relies on multiple paths and how they compare to each other. Therefore, in the next section, damage detection based on a multiple path strategy is presented and assessed under different temperatures.

### 5.4. Damage Detection

Multiple paths are compared to each other using a Damage Index (DI) measure in order to detect the impact damage. The main objective is to detect the damage while avoiding false alarms due to temperature change. The DI used in this study is the Residual Energy:

(7) $$DI_{i,j}=\sum_{k=1}^{n}{\left(S_{i\to j,k}-S_{i\to j,k}^{Ref}\right)}^{2}$$

In order to avoid the panel edge reflections and focus on the scattering close to the direct path, the time window has been set to the actuation period ($\Delta t=n_{cycles}/f$). Although the presented calibration method helps to compensate any changes related to temperature or the acquisition procedure, it does not affect the sensitivity of the Lamb waves to damage. Unlike a conventional guided waves-based SHM system, the FBG sensors are directional and therefore more sensitive along the axis of the fibre optic. In order to consider the calibration procedure successful in temperature changing environment, the states Pristine and Impacted are compared to each other at different temperatures, 20 and 25 ${}^{{}^{\circ}}C$, respectively. The DI of the direct path should be higher than the others, but also the pristine DIs should be significantly lower to avoid false alarm.

**Figure 16:**
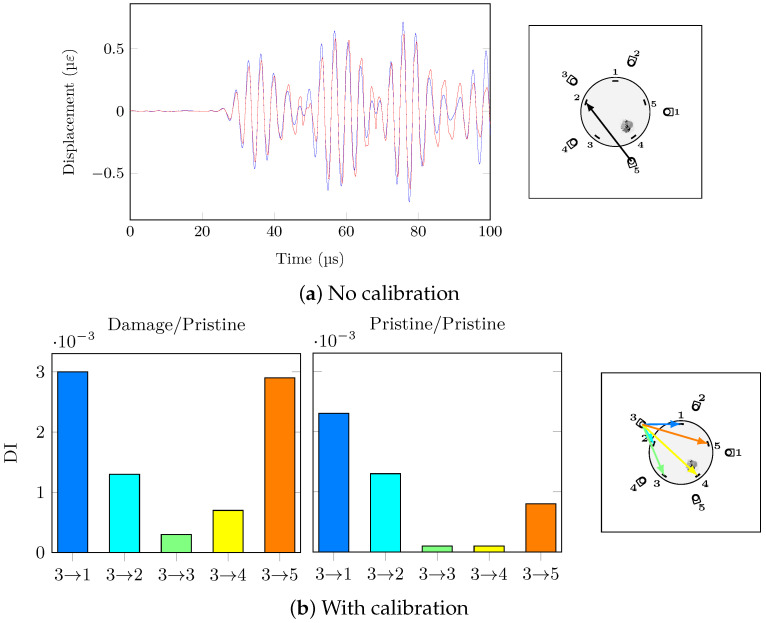
Actuator 3, 60 V 300 kHz, 20 ${}^{{}^{\circ}}C$/25 ${}^{{}^{\circ}}C$, ΔT = 5 ${}^{{}^{\circ}}C$.

Damage detection is frequency dependant. Due to conciseness, only selected frequency response is presented here; more can be found in Appendix B. At low frequencies (below 200 kHz), the A_0_ mode is present, and its out-of-plane particle motion is not sensed by the FBG sensors: their main sensitivity is in-plane along their axis as explained previously. However when the frequency is high enough (300 kHz), S_0_ is the dominant mode and its in-plane motion of particle is easier to detect for the FBG sensors leading to the direct path having the highest DI for an actuation signal at 300 kHz (Figure 16).

**Figure 17:**
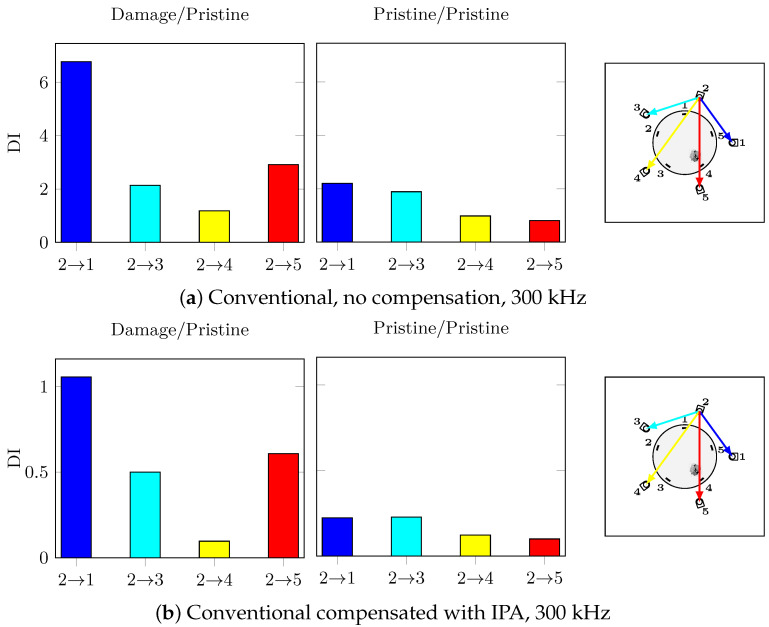
Actuator 2, 60 V, 20 ${}^{{}^{\circ}}C$/25 ${}^{{}^{\circ}}C$, ΔT = 5 ${}^{{}^{\circ}}C$.

Without calibration, the effect of temperature and damage on the hybrid system and the guided waves makes the impact damage undetectable. Furthermore, the DI amplitudes of the damage and pristine cases are similar; hence, the damage detection under temperature variations is impossible with no calibration. On the other hand, with the help of the proposed calibration procedure, the impact damage is correctly detected with the path PZT3→FBG4 having the highest DI at 300 kHz which directly passes through damage. Moreover, the false alarm case of the two different pristine states compared to each other is successfully avoided: the calibration procedure has reduced successfully the effect of temperature in order to highlight the damage effect. In order to confirm that the hybrid system has higher reliability for damage detection for the bonded repair patch under investigation, the conventional paths of actuator PZT2 are shown in Figure 17 at 300 kHz with and without temperature compensation.

As expected, the effect of temperature even after applying compensation on the conventional signals is higher than the hybrid SHM signals. These results emphasise on the complexities of applying SHM to composite step-sanded repair. The impact damage can be considered detected with conventional PZT-PZT system but not located in any of the cases since path 2-5 should have the highest DI value which is not the case.

## 6. Conclusions

In this paper, a hybrid SHM system based on FBG-PZT sensors has been developed for a composite patch repair, to address the challenge of monitoring the integrity of its bondline. To this aim the feasibility of FBG sensors integrated within a composite patch repair has been investigated thoroughly. FBG sensors were embedded in bondline of the last layer and used in hybrid configuration to record guided waves. During the development of the hybrid system, several challenges were highlighted: the change in amplitude of reflectivity was different for each recording due to Bragg peak shift and change of shape, which if not corrected can result in guided wave variations that can be mistaken for damage effect. The novelty of the hybrid set-up proposed in this work was in developing a calibration procedure where the effect of temperature on the FBG spectrum shift has been compensated in addition to compensating the effect of temperature on propagation of the guided wave in the composite repair patch. This novel hybrid acquisition was also tested with experimental results were a step-sanded repair was manufactured and impacted to cause BVID at the bondline. The results of the detection are mixed, in the sense that not all the acquired frequencies were able to detect the damage reliably. However, the higher frequencies where the symmetric mode was dominant (300 kHz) were able to detect damage in the direct path of the PZT-FBG sensors with acceptable reliability. There are many factors that can influence the success of the damage detection for a patch repair. The location of the integrated sensors, the amount of pre-strain in the fibre and the geometry and configuration of the repair patch are just a few factors that can influence the guided wave propagation in the repair patch. The technique has unfortunately drawbacks; the directionality of the FBG sensors makes some paths more damage sensitive than others depending on their respective angle. It was demonstrated that the embedded FBGs have different sensitivities than conventional surface-mounted PZTs for damage and temperature. This is a double-edged sword: while removing the piezoelectric temperature dependency of the PZT sensors, the axial strain sensitivity of the FBGs makes them sensitive to the S_0_ mode and almost totally insensitive to A_0_. This can be problematic for certain damage scenarios, such as in the case of surface damage but, at the same time, is very attractive for local bondline monitoring which has been an ongoing challenge for accepting bonded repair solution for main structural items for aircraft structures.

Therefore, it can be concluded that this work was the first step to thoroughly investigate the feasibility of guided wave application in monitoring and detecting damage in composite patch repair bondline with guided wave. The future work should address the issues of sensorising a composite patch, in order to have reliability and repeatability in the results. In addition, the integrity of the sensors and the bondline should be tested under fatigue load. The main mode of failure for a bonded composite structure is debonding; therefore, the applicability of the SHM system in detecting debonding only should also be investigated.

## Figures and Tables

**Table 1:** Hybrid hardware specifications.

Name	Product	Specifications
AWG	NI PXI-5412	14-bit, 100 MS/s, 12 V${}_{pp}$ output max [50]
Digitizer	NI PXI-5105	12-bit, 8 channels, 60 MS/s, 50 mV${}_{pp}$ to 30 V${}_{pp}$ input range [51]
Switch	40-726A-511-L	12 × 8 RF Matrix Module, 100 VDC max [52]
Chassis	NI PXIe-1073	-
Amplifier	Falco WMA-300	50 Gain, ±150 V max, 2000 $V\;\mu s^{-1}$ slew rate [53]
Laser	Santec TSL-710	1480–1640 nm, ±2 pm accuracy [54]
APD	Thorlabs APD130C/M	900–1700 nm, 0.9 × 10^6^ V/W max [55]
Filter	SRS SIM965	±5 V max, low or high pass analog filter [56]
Switch	OSW12-1310E	1280–1625 nm, 300 mW max [57]

**Figure A2:**
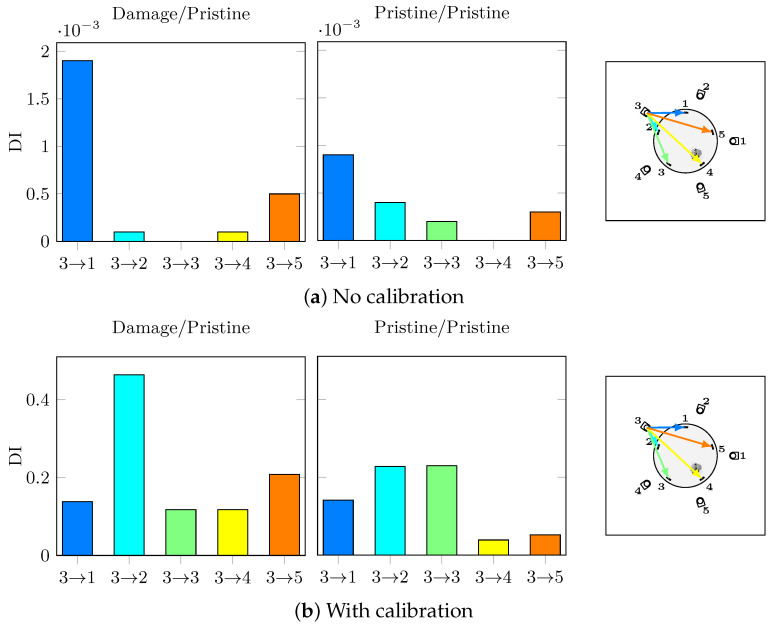
Actuator 3, 60 V 200 kHz, 20 ${}^{{}^{\circ}}C$/25 ${}^{{}^{\circ}}C$, ΔT = 5 ${}^{{}^{\circ}}C$.

**Figure A3:**
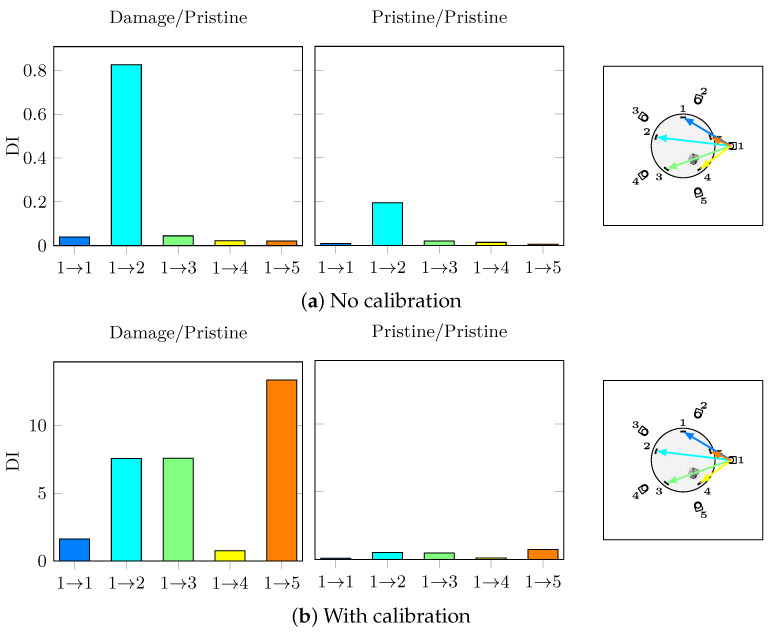
Actuator 1, 60 V 300 kHz, 20 ${}^{{}^{\circ}}C$/25 ${}^{{}^{\circ}}C$, ΔT = 5 ${}^{{}^{\circ}}C$.

## Data Availability

Not applicable.

## Appendix B. Damage Detection Results under Varying Temperature

The results of damage detection with a 5-cycles toneburst of 60 V at 200 kHz with or without calibration are shown in Figure A2. As demonstrated, damage is not detected on the direct path at this frequency.

While some hybrid paths such as path PZT3→FBG4 have successfully detected damage with the proposed calibration procedure, the direction dependency of the embedded fibres is still a major issue. Figure A3 shows the DI charts for actuator PZT1. The calibration procedure correctly enhances the damage detection while reducing the effect of temperature, but PZT1→FBG3 directly goes through damage which is expected to have the highest DI value, but this is not the case. This results highlight the direction dependency of the FO sensors due to the highest sensitivity in their axial direction.
